# Effects of Levetiracetam on Episodic Ataxia Type 2 and Spinocerebellar Ataxia Type 6 with Episodic Ataxic Symptoms: A Case Series

**DOI:** 10.3390/genes16030335

**Published:** 2025-03-13

**Authors:** Haruo Shimazaki

**Affiliations:** 1 Department of Common Education, Faculty of Health and Medical Care, Saitama Medical University, Saitama 350-1241, Japan; hshimaza@saitama-med.ac.jp; Tel.: +81-42-984-4853; 2 Department of Neurology, Jichi Medical University, Tochigi 329-0498, Japan; 3 Department of Neurology, Saitama Medical University, Saitama 350-0495, Japan

**Keywords:** episodic ataxia type 2, spinocerebellar ataxia type 6, *CACNA1A* gene, levetiracetam, acetazolamide

## Abstract

Background: Episodic ataxia type 2 (EA2) is a rare disorder characterized by paroxysmal gait instability, dysarthria, and dizziness. It is caused by *CACNA1A* mutations. Spinocerebellar ataxia type 6 (SCA6) rarely causes episodic ataxia-like symptoms. Acetazolamide has limited effectiveness for treating episodic ataxia. Methods: We investigated the effect of drug therapy in two patients with EA2 and one patient with SCA6 who presented with episodic ataxia. All three cases were CACNA1A-associated diseases. Results: In these three cases, acetazolamide administration was partially and transiently effective for episodic ataxia attacks. After levetiracetam addition, the number of ataxic attacks was significantly reduced, although the durations of attacks were not changed. The effect of levetiracetam was stable and continued for seven years. Levetiracetam and acetazolamide reduced chronic cerebellar ataxia in an SCA6 patient. Conclusions: In this small number of cases, levetiracetam was considered effective in two patients with EA2 and mildly effective in one patient with SCA6.

## 1. Introduction

Episodic ataxia (EA) is a paroxysmal, periodic cerebellar ataxia with no or only mild ataxic symptoms during the interictal period. There are two types of episodic ataxia: inherited and sporadic. The most common hereditary form is episodic ataxia type 2 (EA2) [1]. EA2 is characterized by cerebellar ataxic attacks lasting from a few hours to a few days that exhibit unsteadiness, dysarthria, and vertigo, with nystagmus often present in the interictal period. The *CACNA1A* gene encodes the α1A subunit of the P/Q-type voltage-gated calcium channel (Cav2.1) and is often associated with loss-of-function variants, such as nonsense or frameshift ones [2]. On the other hand, spinocerebellar ataxia type 6 (SCA6) [3], which is caused by an abnormal expansion of CAG repeats in exon 47 of the *CACNA1A* gene, is characterized by slowly progressive cerebellar ataxia, but rarely presents with episodic ataxia-like symptoms [4]. Acetazolamide is often effective for treating EA2 [5] but may be ineffective in some cases. In addition, 4-aminopyridine [6,7] and levetiracetam [8] effectively reduce the attack frequency; however, 4-aminopyridine is currently not available in Japan. In the present study, we report two patients with EA2 and one with SCA6 who presented with episodic ataxia symptoms and were treated with levetiracetam because of an insufficient response to acetazolamide.

## 2. Case Reports

### 2.1. Case 1

**Figure 1:**
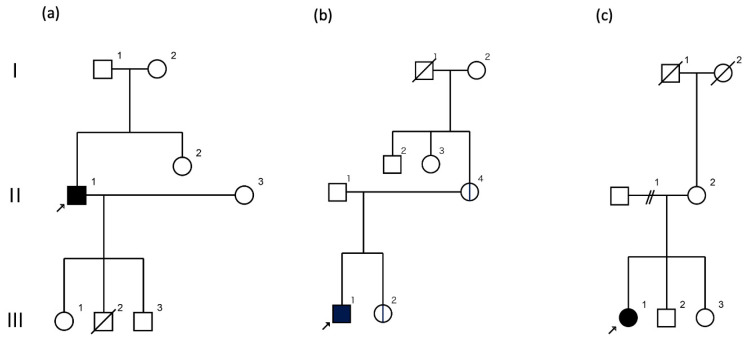
Family trees of the episodic ataxia patients: (**a**) Case 1 (II-1), (**b**) Case 2 (III-1), (**c**) Case 3 (III-1). Cases 1 and 3 had no obvious family history. The family of Case 2 included patients with ataxia (II-4) and absence seizure (III-2) with the same *CACNA1A* variant (p.W1835*).

A 66-year-old male patient had experienced transient unsteadiness attacks, had difficulty using his hands, and had slowed his speech since he was approximately 10 years old. Around the age of 17, he had 2–3 h of stiffness after exercise but recovered after sleep. At 27 years of age, his paroxysmal gait ataxia and dysarthria worsened. The triggers of these attacks included exercise, bathing, mental stress, insomnia, fatigue, coffee consumption, and irritating odors. There was no previous history of disorders or family history of similar diseases, as shown in the family tree (Figure 1a).

At 39 years of age, he visited the Department of Neurology, Jichi Medical University, and was admitted to the hospital for a thorough examination [9]. On admission, there were no abnormal neurological findings other than the presence of lateral gaze nystagmus during the interictal period. After admission, the patient experienced an attack lasting approximately 4 h with slowed speech and ataxia of the trunk and limbs. Blood and cerebrospinal fluid tests were normal, and EEG, head MRI, and MRA showed no abnormalities. The number of CAG repeats for the *CACNA1A* gene was 7/13, which was within the reference range. The patient was discharged from the hospital after administration of acetazolamide (500 mg/day), and his attacks disappeared after 2 years. Thereafter, the patient experienced attacks several times a month, and the acetazolamide dose was increased to 750 mg/day; however, the number of attacks was not reduced.

**Figure 2:**
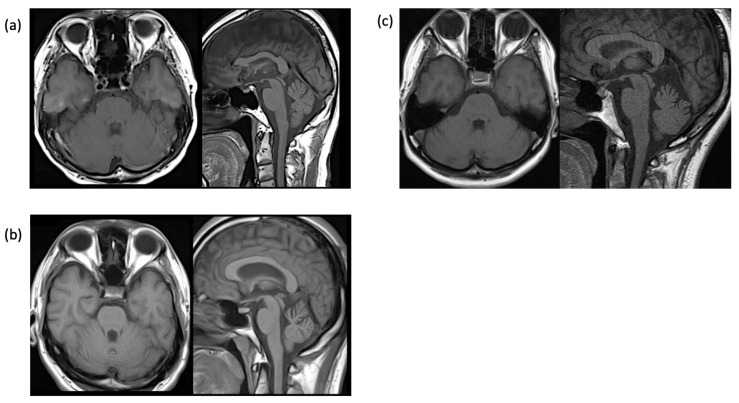
Brain MRI findings of the three patients. (**a**) Case 1, (**b**) Case 2, (**c**) Case 3. T1-weighted axial and sagittal images show mild upper cerebellar vermian atrophy.

**Figure 3:**
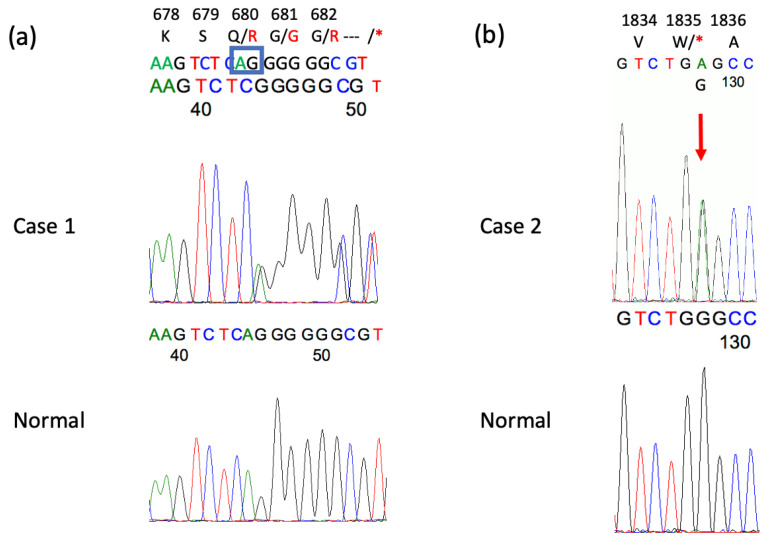
Sequence analyses of *CACNA1A* in Cases 1 and 2. (**a**). A heterozygous 2 bp deletion variant (NM_001127222.2: c.2039-40 del AG (blue box), p.Q680R fs*16) was identified in Case 1. (**b**). A heterozygous nonsense variant (NM_001127222.2: c.5505G>A (red arrow), p.W1835*) was detected in Case 2. This variant was also detected in his mother (Figure 1b II-4) and sister (Figure 1b III-2).

**Figure 4:**
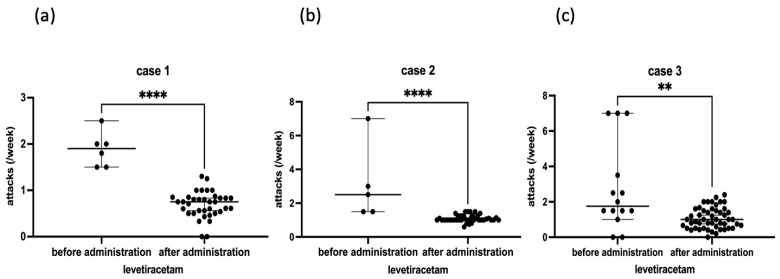
Number of episodic ataxia attacks before and after levetiracetam administration. The number of episodic ataxia attacks was significantly reduced after levetiracetam administration in Case 1 (**a**) and Case 2 (**b**), and slightly reduced in Case 3 (**c**). (Unpaired *t*-test: **** *p* < 0.0001, ** *p* < 0.01.)

At the age of 60 years, the incidence of attacks increased to 2–3 per week and lasted 1–5 h; he revisited our clinic with a referral from another hospital. The electroencephalogram (EEG) revealed intermittent delta waves in the bilateral frontal–temporal regions. The head MRI showed mild cerebellar atrophy (Figure 2a), but the brain SPECT did not show cerebellar hypoperfusion. Whole-exome sequencing identified a previously reported two-base deletion variant (NM_001127222.2: c.2039-40 del AG, p.Q680R fs*16) [10] (Figure 3a). The patient was diagnosed with EA2, levetiracetam was added to acetazolamide starting at 250 mg and then increased to 750 mg, attacks decreased to once every 2 to 3 weeks (Figure 4a), and the duration slightly shortened to 0.5 to 2 h. Currently, attacks occur once every 1–2 weeks and last from 1.5 to 6 h. The effect of levetiracetam was stable for seven years.

### 2.2. Case 2

The proband is a 39-year-old male. Since he was a junior high school student, he has experienced morning attacks of dysarthria lasting about 30 min. The symptoms tended to occur when the temperature rose, when the patient was fatigued, or after a long period of rest. The family history was presented as a family tree (Figure 1b). Although there were no similar cases, the mother experienced mild cerebellar ataxia, and the sister had absence seizures. At the age of 19 years, he visited a university hospital, where he was diagnosed with epilepsy with abnormal electroencephalogram (EEG) and prescribed zonisamide 300 mg/day. However, the drug was not effective, and he stopped visiting the hospital for about six months. He visited the hospital again at the age of 28; his brain MRI findings were unremarkable.

He was referred to our department after resuming treatment with zonisamide 200 mg/day. Neurological examination revealed gaze nystagmus while looking to the left, but no other abnormalities. The head MRI was normal, and EEG showed frequent bilateral frontal spikes and waves. At the age of 30, he stopped taking his medication and visiting our hospital, but at the age of 31, he returned to the hospital because of unsteadiness lasting 1–1.5 h every morning. MRI showed mild cerebellar atrophy (Figure 2b), and brain SPECT showed mild cerebellar hypoperfusion predominantly in the left side. EEG revealed spike and wave complexes and intermittent theta waves in the bilateral frontal regions. Genetic testing with informed consent showed that the number of CAG repeats in the *CACNA1A* gene was within the normal range of 12/13, but whole-exome sequencing identified a novel nonsense variant of the *CACNA1A* gene (NM_001127222.2: c.5505 G>A, p.W1835*) (Figure 3b). The same variant was found in his mother and sister. He was diagnosed with EA2 and started acetazolamide 500 mg/day at 32 years of age.

After initiating acetazolamide, daily attacks were reduced to 1 to 3 times a week. At 33 years of age, we started adding 500 mg of levetiracetam, and the dose was increased to 1000 mg. The current attack frequency is approximately once a week (Figure 4b), and the duration is approximately 1–3 h. The effect of levetiracetam was stable for seven years.

### 2.3. Case 3

A 46-year-old woman presented with episodic ataxia during sitting and walking, with a frequency of approximately once a month since the age of 24 years, which improved within 2 to 3 h. She tended to have attacks when under mental stress. There was no family history as shown in the family tree (Figure 1c). At 27 years of age, her attacks increased to about once a week, and she was admitted to the Department of Neurology, Jichi Medical University Hospital, for a thorough examination [11]. On admission, neurological examination revealed no nystagmus or other neurological findings during the interictal period. During hospitalization, three attacks occurred. During the attacks, bilateral gaze nystagmus, slurred speech, truncal ataxia, and gait instability were observed, and the symptoms persisted for about 2.5 h. Blood, cerebrospinal fluid, and electroencephalography findings were normal, and head MRI revealed a mildly enlarged folia on the superior cerebellar vermis (Figure 2c). Brain SPECT showed no cerebellar hypoperfusion, and ^1^H-MR spectroscopy showed a decreased NAA/Cr ratio in the cerebellar hemispheres. Informed consent was obtained for genetic testing of *CACNA1A*, which showed no pathologic nucleotide substitutions, but CAG repeats were expanded to 22/13. The patient was diagnosed with SCA6 with episodic ataxia.

She started taking acetazolamide 500 mg/day at 29 years of age after delivery and lactation at her request, and a mild decrease in attack frequency was observed. Thereafter, attacks continued to occur once a week to almost every day, with durations ranging from 1 h to half a day. The acetazolamide dose was increased to 750 mg/day without any change, and levetiracetam was added at age 41 at 250 mg/day and titrated to 1000 mg/day. The attack frequency ranged from twice a week to once every two weeks (Figure 4c), and the duration was approximately 1–7 h. The effect of levetiracetam was stable for seven years.

At 43 years of age, the patient developed persistent trunk ataxia and gait ataxia. At 45 years of age, after self-withdrawal of acetazolamide and levetiracetam for about 1 month without visiting the clinic, her trunk and gait ataxia worsened (SARA score: 18 points), but returned to the basic level (SARA score: 8 points) when she resumed taking the medication.

## 3. Discussion

The clinical findings in the three episodic ataxia patients are shown in Table 1. In this study, three patients with episodic ataxia were treated with levetiracetam additionally because of an inadequate response to acetazolamide, resulting in a decrease in attack frequency. The efficacy of levetiracetam against EA2 was previously reported in some cases [8,12].

Acetazolamide is mentioned to be effective in 50–75% of patients with EA2 by regulating neuronal firing through its pH regulation. The effect of acetazolamide may decrease after long-term use [13]. In the three patients in this study, acetazolamide was transiently or only mildly effective, and levetiracetam was added with the patients’ consent, resulting in a further reduction in attack frequency.

EA2 is believed to be caused by the loss of Cav2.1 function due to nonsense or frameshift variants in the *CACNA1A* gene [14]. A so-called dominant–negative mechanism, in which the mutant protein inhibits the function of the normal protein, has also been postulated [15]. SCA6 is also thought to be caused by a dominant–negative mechanism; an aberrant long C-terminal polyglutamine stretch is generated from an aberrant gene with CAG repeat expansion, and the C-terminal portion is detached by alternative splicing or post-translational processes, causing the polyglutamine chain to accumulate in the cytoplasm and nucleus, inhibiting gene expression [16].

There are several triggers for episodic ataxia attacks in EA2. In Case 1, attacks were triggered by the consumption of coffee and exercise, which increased body temperature. The caffeine included in coffee activates ryanodine receptors on the surface of the endoplasmic reticulum (ER) [17], releasing calcium stored in the ER of Purkinje cells into their cytoplasm and increasing the intracellular calcium concentration [18]. The increase in intracellular calcium stimulates the release of neurotransmitters from Purkinje cells and suppresses the activity of cerebellar nuclei in the postsynaptic region, which may result in ataxic attacks [19]. In addition, elevated body temperature increases calcium influx from the extracellular to the intracellular space via temperature-sensitive transient receptor potential (TRP) channels on the Purkinje cell surface, which is believed to induce ataxic attacks by the same mechanism.

Levetiracetam binds to synaptic vesicle glycoprotein 2A (SV2A) and exerts its antiepileptic effect by inhibiting presynaptic calcium channels via an intracellular pathway and significantly inhibiting caffeine-induced intracellular calcium ion release via ryanodine receptors in a concentration-dependent manner [20].

These mechanisms suggest that levetiracetam suppresses abnormal neurotransmitter release from Purkinje cells by inhibiting intracellular calcium release, which may reduce ataxic attacks. However, levetiracetam has many actions, such as decreasing the function of SV2A, modulating synaptotagmin protein expression and traffic, reducing potassium currents, modulating GAD, increasing GABA transaminase, blocking GABA_A_ receptors, and modulating AMPA receptors [17]. It remains possible that other mechanisms may be responsible for its efficacy.

Case 2’s mother and sibling had the same nonsense variant in the *CACNA1A* gene, but they showed clinical heterogeneity with different symptoms: the mother had chronic cerebellar ataxia, Case 2 had episodic ataxia, and his sibling had absence seizures. Several such families have already been reported [21,22].

Case 3 was a patient with SCA6 with episodic ataxia who developed chronic cerebellar ataxia over time. After accidental self-interruption of levetiracetam and acetazolamide for about a month, chronic cerebellar ataxia symptoms worsened but returned to the previous ataxia level upon resumption of the medication. Levetiracetam and acetazolamide may be effective for treating chronic cerebellar ataxia, but further studies with a larger number of patients are needed.

## 4. Conclusions

In this study with a small number of cases, we observed a reduction in the number of episodic ataxias in three patients with *CACNA1A* variants following the addition of levetiracetam to acetazolamide. Levetiracetam was more effective in reducing the frequency for EA2 than SCA6. Levetiracetam could be useful for episodic ataxia treatment. An additional study with a large number of cases would elucidate the levetiracetam effect for episodic and chronic ataxias.

## Figures and Tables

**Table 1:** Clinical findings in three episodic ataxia patients.

	Case 1	Case 2	Case 3
Onset	10 y	13 y	24 y
Age at examination, gender	66 y, M	39 y, M	46 y, F
Body weight (kg)	57	70	52
Race	Asian	Asian	Asian
Interictal nystagmus	+	+	−
Ictal duration	30 min–12 h	10–30 min	2–3 h
Ictal symptoms (IS): dysarthria	+	+	+
IS: Limb ataxia	+	+	−
IS: Truncal ataxia	+	+	+
Migraine	−	+	−
EEG	intermittent delta activity	spike and wave, intermittent theta activity	normal
Brain MRI: cerebellar atrophy	+−	+	+
Brain SPECT: cerebellar blood flow	→	↓	→
*CACNA1A* gene variant/amino acid change	c.2039_2040delAG/p.Q680R fs*16	c.5505G>A /p.W1835*	−
CAG repeat numbers in *CACNA1A* gene	7/13	12/13	13/22
Acetazolamide effect	+	+−	+−
Maximum levetiracetam dose (mg/day)	750	1000	1000
Levetiracetam effect	++	++	+

## Data Availability

The original contributions presented in this study are included in the article. Further inquiries can be directed to the corresponding author.
